# Identifying behaviour change techniques in school-based childhood obesity prevention interventions: a secondary analysis of a systematic review

**DOI:** 10.1186/s12889-025-23421-9

**Published:** 2025-07-02

**Authors:** Daniel C.W. Lee, Serene Yoong, Sam McCrabb, Brittany J. Johnson, Justin Presseau, Ashleigh Stuart, Kate M. O’Brien, Rebecca K. Hodder

**Affiliations:** 1 Hunter New England Population Health, Hunter New England Local Health District, Wallsend, Australia; 2 National Centre of Implementation Science, University of Newcastle, Newcastle, Australia; 3 College of Health Medicine and Wellbeing, University of Newcastle, Newcastle, Australia; 4 Hunter Medical Research Institute Population Health Program, Newcastle, Australia; 5 Global Centre for Preventive Health and Nutrition, Deakin University, Burwood, Australia; 6 Flinders University, College of Nursing and Health Sciences, Caring Futures Institute, Bedford Park, Adelaide, 5042 Australia; 7 Methodological and Implementation Research Program, Ottawa Hospital Research Institute, Ottawa, Canada; 8 School of Epidemiology and Public Health, University of Ottawa, Ottawa, Canada; 9 Hunter Medical Research, Newcastle, Australia

**Keywords:** Childhood obesity prevention, Behaviour change techniques, Healthy eating, Physical activity

## Abstract

**Background:**

Childhood overweight and obesity is increasingly prevalent, can persist into adulthood, and lead to lifelong negative health trajectories. Schools are a recommended setting for childhood obesity prevention interventions; however, these interventions are often complex and multicomponent. While previous reviews have demonstrated their effectiveness, they have not identified which behaviour change techniques (BCTs – active ingredients of an intervention) are most effective.

**Objectives:**

Describe BCTs used in healthy eating (HE) and physical activity (PA) intervention components of obesity prevention interventions supporting children aged 6–18 years; and explore which BCTs are associated with child weight.

**Methods:**

A secondary analysis of school-based trials included in a 2022 update of a Cochrane systematic review was undertaken. The previous review included 195 randomised controlled trials of childhood obesity prevention interventions targeting HE and/or PA that assessed the body mass index of children aged 6–18 years. For this study, only trials delivered in schools that compared an intervention to a non-intervention control group and targeted HE, PA or both were eligible. Individual BCTs of each HE and PA intervention were coded according to the BCT taxonomy v1. Meta-regressions were conducted to determine the association between BCTs included in the trials and child weight.

**Results:**

This secondary analysis included 124 eligible trials. Fifty-five of the 93 BCTs from 14 of the 16 BCT domains were identified across interventions. Interventions with a HE component that included BCTs from three domains (*Goals and planning; Social support; Comparison of behaviour*) were found to have a significant association with a positive effect on child weight, whereas there were no significant associations found for interventions with a PA component.

**Conclusion:**

School-based obesity prevention interventions with HE components that included BCTs within the *Goals and planning, Social support,* and *Comparison of behaviour* domains, such as *Goal setting (outcome)*, *Social support (unspecified)* and *Demonstration of the behaviour* were associated with a positive effect on child weight and should be considered for prioritisation in future interventions. Further research is required to identify effective BCTs for PA intervention components, and for effective individual BCTs and combinations of BCTs for all obesity prevention interventions broadly.

**Trial registration:**

CRD42022366743.

**Supplementary Information:**

The online version contains supplementary material available at 10.1186/s12889-025-23421-9.

## Introduction

### Background

Obesity is the leading contributor of non-fatal burden of disease and the second leading risk factor for total burden of disease [1]. A person with overweight or obesity is at a higher risk of developing many serious health conditions, such as cancer and cardiovascular disease. In 2017, obesity was responsible for 4.7 million premature deaths globally [2] and cost the economy approximately 2.19% of the global gross domestic product in 2022 [3]. There is a growing prevalence of overweight and obesity, with more than 340 million children worldwide categorised as overweight or obese in 2016 [4], an increase of 5.6% for girls and 7.8% for boys aged 5–19 years since 1975 [5].

To address the obesity epidemic, the World Health Organisation (WHO) and other research organisations have stated that poor diet (e.g. inadequate consumption of fruits and vegetables) and physical inactivity are key determinants of obesity [6, 7]. Poor diet and physical inactivity are health risk behaviours that lead to childhood obesity, are often established in childhood and track into later life [8]. Strategies are therefore recommended to prevent obesity and reduce the associated burden of disease [9, 10]. Schools are a key recommended setting for obesity prevention efforts because of the large potential impact they can have due to their existing infrastructure, resources and access to large populations of children [11, 12] at a stage of life where they form lifelong health behaviour habits [13]. For example, in Australia, children spend up to approximately 30 h per week in schools [12].

There has been numerous trials and investment globally in developing and evaluating school-based obesity prevention interventions that target healthy eating (HE) and physical activity (PA) to identify effective evidence-based interventions. High-quality reviews of randomised controlled trials (RCTs) synthesising the results of these programs found them to be generally effective in preventing child obesity [11, 14, 15]. A 2021 update of a Cochrane systematic review that focussed on PA interventions found evidence of very small decrease in body mass index (BMI) z-scores (mean difference −0.06, 95% confidence interval (CI) −0.09 to −0.02; 21 trials, n = 22,948) [11]. Another Cochrane review that assessed the effectiveness of childhood obesity prevention interventions on the weight of children, found positive, albeit small, effect on BMI/BMI z-scores (standardised mean difference (SMD) −0.03, 95% CI −0.06, −0.01; 93 trials, n = 131,443) [16]. While the findings of such reviews provide overall evidence of the positive impact of school-based obesity prevention programs, by design, they typically pool studies to determine overall effectiveness across a group of similar trials. This is a particular issue with school-based obesity prevention programs as they are predominantly multicomponent, complex, and heterogeneous and such reviews typically do not examine the effectiveness of individual intervention components [16].

While there are many approaches to understanding effective individual components, including but not limited to, non-randomised study designs, network meta-analysis and qualitative comparative analysis, one promising approach is the characterisation of behaviour change techniques (BCTs). BCTs are defined as planned processes that are the smallest active ingredient in an intervention, which can be used alone, or in combination [17]. BCTs are theorised to be the core active ingredient (e.g. *Instructions on how to perform the behaviour*) that through mechanisms of actions (e.g. increasing knowledge, skills and beliefs about capabilities) results in behaviour change (e.g. increased physical activity) which addresses the determinants of behaviour (e.g. capability) [18, 19]. The identification of the BCTs included in interventions allows for better description of interventions [20], better understanding of the discrete effective components of interventions, and also the ability to synthesise and, across a body of research, assess which BCTs are effective [21–23]. For example, a study identified effective BCTs in a smoking cessation program [24] and used these BCTs to guide the development of a training course that has been found to be associated with increased success rate of smoking cessation [25]. Taxonomies of BCTs help describe and categorise BCTs in a standardised manner that allows for comparison among similar research fields. The most comprehensive and widely used taxonomy internationally is the Behaviour Change Technique Taxonomy version 1 (BCTTv1), which contains 93 BCTs across 16 domains and has proven useful in categorising and defining BCTs [20, 26], including in childhood obesity prevention [27] and is the taxonomy we adopted for this study.

A number of previous studies have been conducted to identify effective BCTs of obesity prevention interventions [20, 22, 23, 28–35]. For example, a prospectively registered review (2017) of 48 trials (n = 11,183 participants) which aimed to identify which BCTs were effective in HE and PA interventions in adults with obesity [23]. Of the 29 BCTs (identified using the BCTTv1 taxonomy), two (goal setting of behaviour and self-monitoring of behaviour) were associated with a positive intervention effect on adult weight in meta-regression [23]. A mixed-methods study conducted in 2020 found that BCTs included in HE and PA interventions in four similar early childhood obesity prevention trials (up to 18 months postnatal) [29] reported use of 35 distinct BCTs using the BCTTv1 taxonomy but did not examine which of these were associated with an impact on reducing excessive child weight gain [29]. Another study (under review) sought to characterise effective BCTs in early childhood (antenatal to 12 months postnatal) and found 49 unique BCTs were coded to at least one target behaviour (infant (milk) feeding, food provision and parent feeding, movement and sleep health) [35]. No previous studies could be identified that explored which BCTs included in obesity prevention interventions supporting children aged 6–18 years were associated with an effect on child weight.

### Objectives

This study objectives are to; 1) describe BCTs used in HE and PA intervention components of school-based obesity prevention interventions supporting children aged 6–18 years and; 2) explore which a) BCT domain, b) individual BCTs and c) discrete use of individual BCTs or combinations of BCTs are associated with child weight.

## Methods

This study is a secondary data analysis of trials included in a 2022 update of a Cochrane systematic review of obesity prevention interventions, in children aged 6–18 years [16]. The protocol was prospectively registered with PROSPERO (CRD42022366743).

### Eligibility criteria

Briefly, as per the eligibility criteria outlined in the previous review [16], RCTs that compared a child obesity prevention intervention with either a 1) non-intervention or usual care control group, or 2) an alternate intervention, in any setting were included. Trial participants were children with a mean age between six and 18 years at baseline (school-aged children). Trial interventions had to have a rationale to prevent child obesity, and report an eligible child weight outcome (BMI, BMI z-scores, prevalence of overweight and/or obesity, weight, percent fat content, and skin-fold thickness) at baseline and post-intervention at least 12 weeks from baseline (irrespective of intervention duration). While all weight outcomes were eligible, only BMI/BMI z-score were synthesised in meta-analysis.

Trials that only enrolled children who were overweight or obese, sampled children with critical illness or severe co-morbidities, designed to treat (rather than prevent) childhood obesity or eating disorders, and those focused solely on strength and fitness training (rather than to increase time in PA for preventing obesity), or that included any drug, supplement, or surgical intervention, were excluded.

Additionally, to be included in this secondary data analysis, trials were required to have been primarily conducted in either primary or secondary school setting (i.e. after-school program, community, home, health care services were excluded), compared an intervention against a non-intervention control group (i.e. alternate intervention or comparative effectiveness trials were excluded) and target either HE (e.g. nutrition education), PA (e.g. physical activity session) or both (i.e. reporting measured BMI as an intervention were excluded).

### Search methods

The previous review identified eligible trials between 1990 to June 2021 via a comprehensive search of electronic databases (Cochrane Central Register of Controlled Trials (CENTRAL), Medline, Embase, Cumulative Index to Nursing and Allied Health Literature (CINAHL) and PsycINFO), trial registries, and reference lists and contact with authors of included trials [16]. An additional author search, which involved searching for trial registries, protocols and associated papers was conducted for this review for trials with limited intervention details for coding BCTs.

### Screening

Two authors (DL, RH) independently screened the full texts of all trials included in the existing review against the updated eligibility criteria described above, with any discrepancies resolved via discussion or by a third reviewer where required (SY).

### Data extraction

Pairs of authors from the previous review independently extracted trial characteristics, including study design, participants, targeted behaviours (HE, PA, or both), and weight outcomes, with priority given to first follow-up and BMI z-score over BMI for synthesis. All data was sourced from the previous review [16].

### Coding of BCTs

The BCTs identified within the HE and PA intervention components of all included trials were coded using the BCTTv1 (containing 93 BCTs across 16 domains) [26] according to standardised protocol. The 16 groups within the BCTTv1 categorize similar BCTs into single domains (e.g. social support, repetition and substitution) [26]. The protocol involved: 1) coders reading the intervention description within the primary paper and any other associated papers including trial protocols, and additional materials; and 2) using the taxonomy as a checklist to confirm the presence of individual BCTs. A codebook was developed to provide coders with clarifications around common discrepancies of BCTs and frequently used examples that are relevant to HE and PA interventions, which was updated throughout the course of extraction and reviewed by SY (Supplementary file 1). If the included trials indicated that BCTs were used to design the intervention, and these were reported by authors, we coded the BCTs based on the BCTTv1 or earlier versions.

Coders were required to complete the BCTTv1 training and achieve competence [36] and attend a training session on the standardised protocol. Coding was entered directly into REDCap [37, 38], an electronic data capture tool, hosted at Hunter New England Local Health District. The coding process was first piloted on the included trials by two coders independently (DL, SM), in an alphabetical order for the first 20% [39]. Inter-rater reliability was then calculated on the next 10% of trials at a domain level. The two coders met weekly to consolidate, discuss discrepancies and update the codebook. Any discrepancies between coders were resolved via consensus or by a third author (SY). After achieving reasonable agreement of 81% [40], the remaining trials (k = 95) were single coded by one coder (DL).

### Analysis and synthesis

All statistical analyses were performed in R version 4.3.2 [41] by one author (AS).

#### Objective 1

Describe the trial characteristics and BCTs used.

Only trials that reported BMI/BMI-z score were synthesised in the previous review and therefore considered for the meta-regression for this study. The characteristics of trials (including BCT use) that did not report BMI/BMI z-scores, are described narratively. Descriptive statistics (percent and sample size) were used to describe the targeted behaviours (HE and/or PA), trial characteristics, and BCTs used in school-based obesity prevention interventions.

#### Objective 2

Explore which a) BCT domain, b) individual BCTs and c) discrete use of individual BCTs or combinations of BCTs are associated with change in child weight. This association could be positive (reducing excessive weight gain in children, i.e. preventing obesity) or negative (excessive weight gain in children, i.e. increasing risk of obesity).

Random effects meta-regression models with restricted maximum likelihood estimation and Knapp-Hartung variance estimators were used to examine the effect of BCTs on child BMI/BMI z-scores using the R package ‘metafor’ [42]. Random effects were included as it was expected that the true intervention effects would vary after accounting for the effect of BCTs. Models included a) BCT domains, b) individual BCTs, or c) discrete use of individual BCTs or combinations of BCTs in intervention and/or control groups as moderators to examine their effects on BMI/BMI z-scores in both HE and PA interventions separately. Dummy variables were included to indicate whether an intervention included a) BCTs within a domain, b) individual BCTs or c) discrete use of individual or combinations of BCTs. Where a BCT was used in both the intervention and control group, it was not coded as included in the intervention and control group as it was not considered responsible for a difference in effect.

#### Objective 2a and 2b

For BCTs to be included as moderators they must have been used in ten or more interventions, per the Cochrane handbook [43]. Analysis of BCT domains and individual BCTs included all included trials irrespective of whether any BCTs were coded.

#### Objective 2c

To be included in the discrete use of individual BCTs or combinations of BCTs analysis, a minimum of two trials were required. Analysis of discrete individual BCTs or combinations of BCTs only included trials that belonged to each of the discrete BCTs.

Results are presented as the estimated average SMD and 95% CI for each moderator, adjusted for the effects of the other moderators, with an omnibus test of moderator differences. The SMD was calculated from BMIs or BMI z-scores, therefore a negative SMD suggests a reduction in BMI and a more effective intervention. Heterogeneity was measured using *I*^2^ and *Tau*^2^ [44] and the direction and magnitude of SMD were examined and reported.

While the meta-regressions allowed interventions to use different combinations of BCTs, they did not allow for interactions between them. To further address the question of which combination of BCTs is the most effective, meta-CART was used to identify any potential interactions between BCTs using the R package ‘metacart’ [45]. Meta-CART involves identifying more homogenous subgroups with respect to their effect sizes using a classification and regression tree (CART) then performing subgroup meta-analysis. The CART process examines the between subgroup heterogeneity when interventions are split into subgroups based on the presence/absence of a moderator (when categorical). It examines all possible options before picking the split that maximises the between subgroup heterogeneity. This process is then repeated, forming a tree, until stopping criteria is reached. The stopping criteria includes a threshold for the minimum between subgroup heterogeneity or too few trials in a group to split any further. The tree is then pruned to prevent overfitting. Random effects meta-analysis using the DerSimonian-Laird estimator then compares the effect sizes between subgroups [46]. As this analysis was exploratory, a minimum terminal node size (i.e. number of BCTs in the final subgroups) of two and a pruning parameter (i.e. cut-off to prevent overfitting) of zero were selected.

## Results

### Search result and trial characteristics

**Fig. 1:**
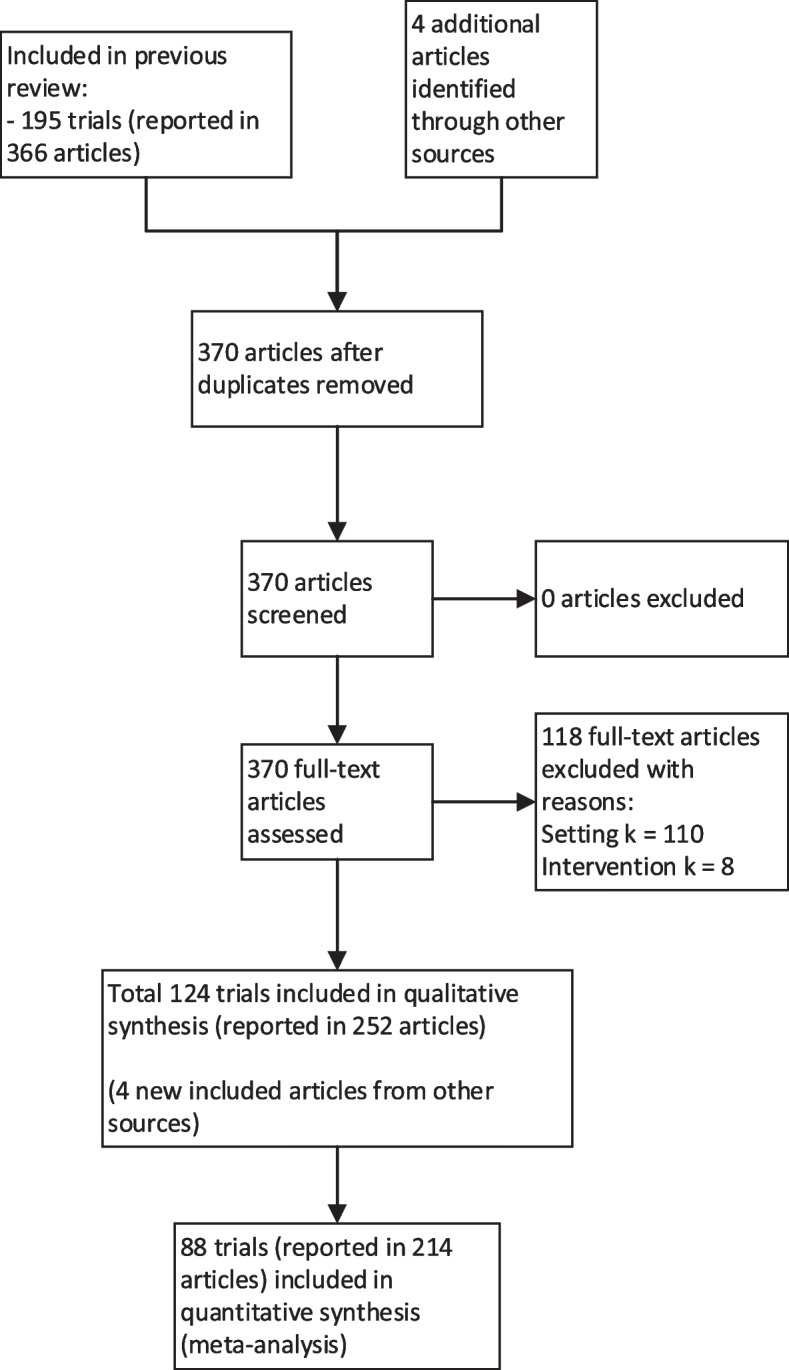
PRISMA flow diagram

All 195 trials (from 366 records) from the previous review were screened against the updated eligibility criteria [16]. In total, 124 trials (from 252 records) met the updated eligibility criteria and were included in the current study (Fig. 1, Supplementary file 2). Four additional records were identified as associated papers reporting on included trials though the additional author search.

There were 275 trial arms and 146,541 participants included in the 124 trials, of which 13 were RCTs, and 111 were cluster-RCTs. Most trials were conducted in high-income countries (77%, k = 95), with the majority conducted in the United States of America (24%, k = 30), followed by Australia (9%, k = 11), China (8%, k = 10) and United Kingdom (8%, k = 10).

Most trials included children aged 6–12 years (74%, k = 92), and tested interventions targeting both HE and PA (61%, k = 76). There were 92 HE intervention components and 108 PA intervention components from all trials. Trials ranged in duration from two months to four years, with the majority 12 months or less in duration (71%, k = 88).

BMI/BMI z-score data were available for 107 trials (86%), of which 19 trials were unable to be synthesised in the meta-regression of the current study; 18 due to insufficient data for synthesis and one due to flexibility of intervention, resulting in inability to reliably code BCTs. Seventeen trials reported other weight outcomes; five prevalence of overweight and/or obesity, four BMI percentile, three percentage body fat, two fat mass and/or weight, one waist circumference, one underweight vs obesity and one a combination of other weight outcomes. The first follow-up point greater than 12 weeks post baseline ranged from 12 weeks to two years.

### Aim 1: BCTs included by included trials

Of 93 possible BCTs in the taxonomy, a total of 55 BCTs were coded to the interventions in at least one trial (Supplementary file 3). The number of BCTs per trial ranged from zero (unable to be coded due to insufficient information, k = 3) to 18, with an average of 5.4 BCTs per trial.

Individual BCTs were identified across 14 of the 16 BCT domains with the most frequently coded domain among HE intervention components, *Shaping knowledge* (20%, n = 84) and among PA intervention components, *Repetition and substitution* (18%, n = 99) (Table 1). No BCTs were identified in domains *Scheduled consequences* and *Covert learning*.

**Table 1:** Number of BCTs coded in each domain from HE and PA trials (k = 124)

	**Domains** *(number of individual BCT in each domain)*	**HE intervention components** **n (%)**	**PA intervention components** **n (%)**
1	Goals and planning (*n* = 9)	55 (13%)	76 (14%)
2	Feedback and monitoring (*n* = 7)	38 (9%)	66 (12%)
3	Social support (*n* = 3)	12 (3%)	19 (3%)
4	Shaping knowledge (*n* = 4)	84 (20%)	88 (16%)
5	Natural consequences (*n* = 6)	20 (5%)	12 (2%)
6	Comparison of behaviour (*n* = 3)	34 (8%)	48 (9%)
7	Associations (*n* = 8)	10 (2%)	8 (1%)
8	Repetition and substitution (*n* = 7)	55 (13%)	99 (18%)
9	Comparison of outcomes (*n* = 3)	22 (5%)	16 (3%)
10	Reward and threat (*n* = 11)	21 (5%)	31 (6%)
11	Regulation (*n* = 4)	5 (1%)	4 (1%)
12	Antecedents (*n* = 6)	60 (14%)	64 (12%)
13	Identity (*n* = 5)	7 (1%)	15 (2%)
14	Scheduled consequences (*n* = 10)	0 (0%)	0 (0%)
15	Self-belief (*n* = 4)	4 (1%)	5 (1%)
16	Covert learning (*n* = 3)	0 (0%)	0 (0%)

The most frequently coded individual BCTs in HE and PA intervention components were *Instruction on how to perform the behaviour* (HE: 19%, n = 82; PA: 16%, n = 87) and *Behavioural practice/rehearsal* (HE: 10%, n = 41; PA: 15%, n = 81) (Table 2 and 3). There were 38 BCTs that were not identified in the included trials. Three HE intervention components and two PA intervention components had no identifiable BCTs.

**Table 2:** Ten most frequently coded individual BCTs in all HE intervention components (n = 92)

	**BCTs**	**HE intervention components** **n (%)**
4.1	Instruction on how to perform the behaviour	82 (19%)
8.1	Behavioural practice/rehearsal	41 (10%)
12.1	Restructuring the physical environment	29 (7%)
12.5	Adding objects to the environment	28 (7%)
6.1	Demonstration of the behaviour	28 (7%)
9.1	Credible source	20 (5%)
1.3	Goal setting (outcome)	15 (4%)
1.2	Problem solving	15 (4%)
5.1	Information about health consequences	14 (4%)
2.3	Self-monitoring of behaviour	13 (3%)

**Table 3:** Ten most frequently coded individual BCTs in all PA intervention components (n = 108)

	**BCTs**	**PA intervention components** **n (%)**
4.1	Instruction on how to perform the behaviour	87 (16%)
8.1	Behavioural practice/rehearsal	81 (15%)
12.5	Adding objects to the environment	41 (7%)
6.1	Demonstration of the behaviour	38 (7%)
2.3	Self-monitoring of behaviour	26 (5%)
1.3	Goal setting (outcome)	25 (4%)
12.1	Restructuring the physical environment	17 (3%)
1.2	Problem solving	16 (3%)
3.1	Social support (unspecified)	16 (3%)
2.2	Feedback on behaviour	15 (3%)

### Aim 2a: Meta-regression analysis of BCT domains and child weight

#### Healthy eating

**Fig. 2:**
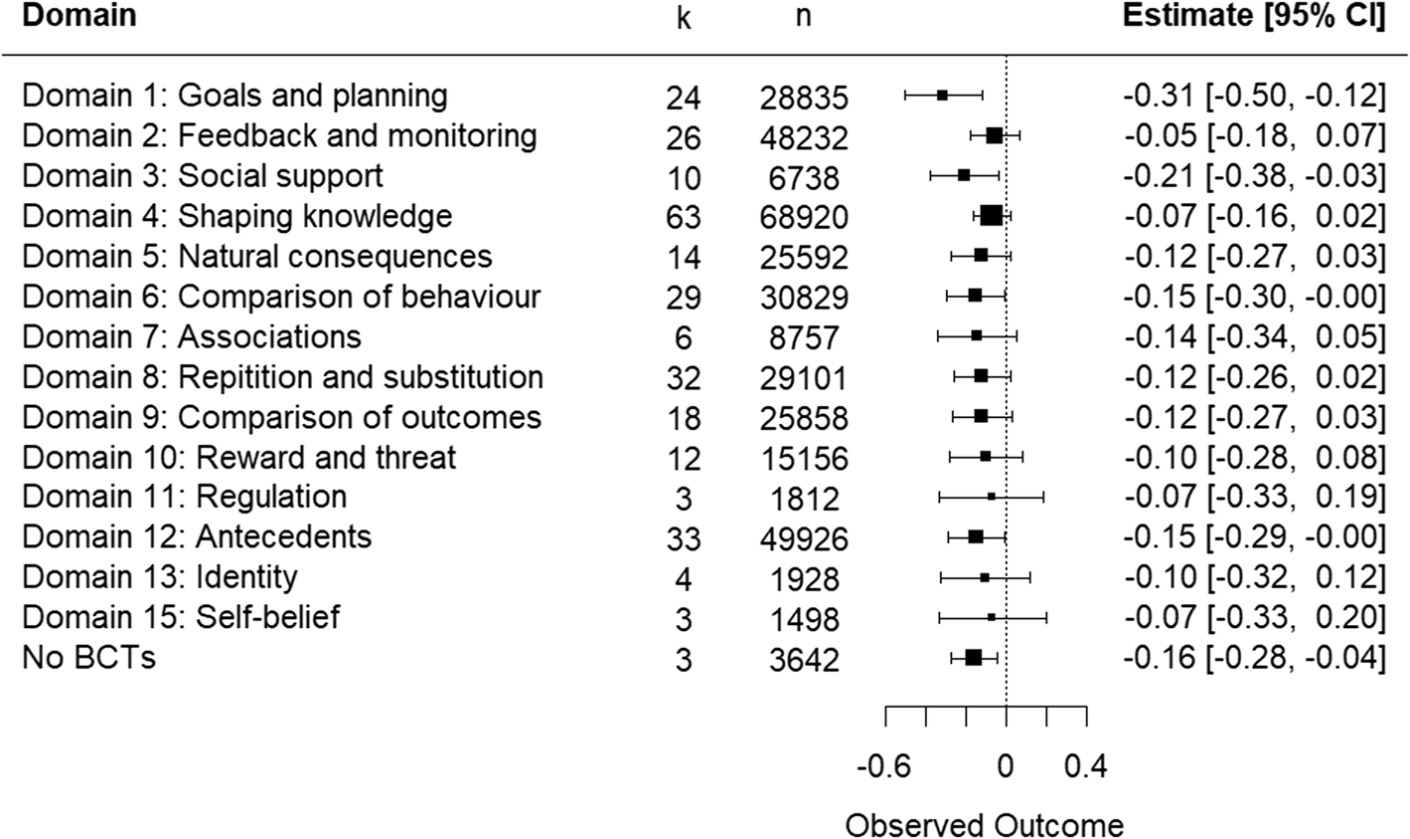
Meta-regression showing effect of BCT domains in healthy eating interventions on child weight

Meta-regression of 67 HE intervention components (n = 92,256 participants) comparing BCT domains revealed domains 1, 3 and 6 had a statistically significant association with a positive effect on child weight (Domain 1: SMD −0.311, 95% CI −0.383, −0.029; Domain 3: SMD −0.206 95%, CI −0.383, −0.029; Domain 6: SMD −0.150, 95% CI: −0.298, −0.001; Fig. 2). A test of moderator difference showed no differences in effect between domains (Q_m_ = 0.89, I^2^ = 70.53%; Fig. 2). The heterogeneity was high (I^2^ = 66.88%, Tau^2^ = 0.01).

#### Physical activity

**Fig. 3:**
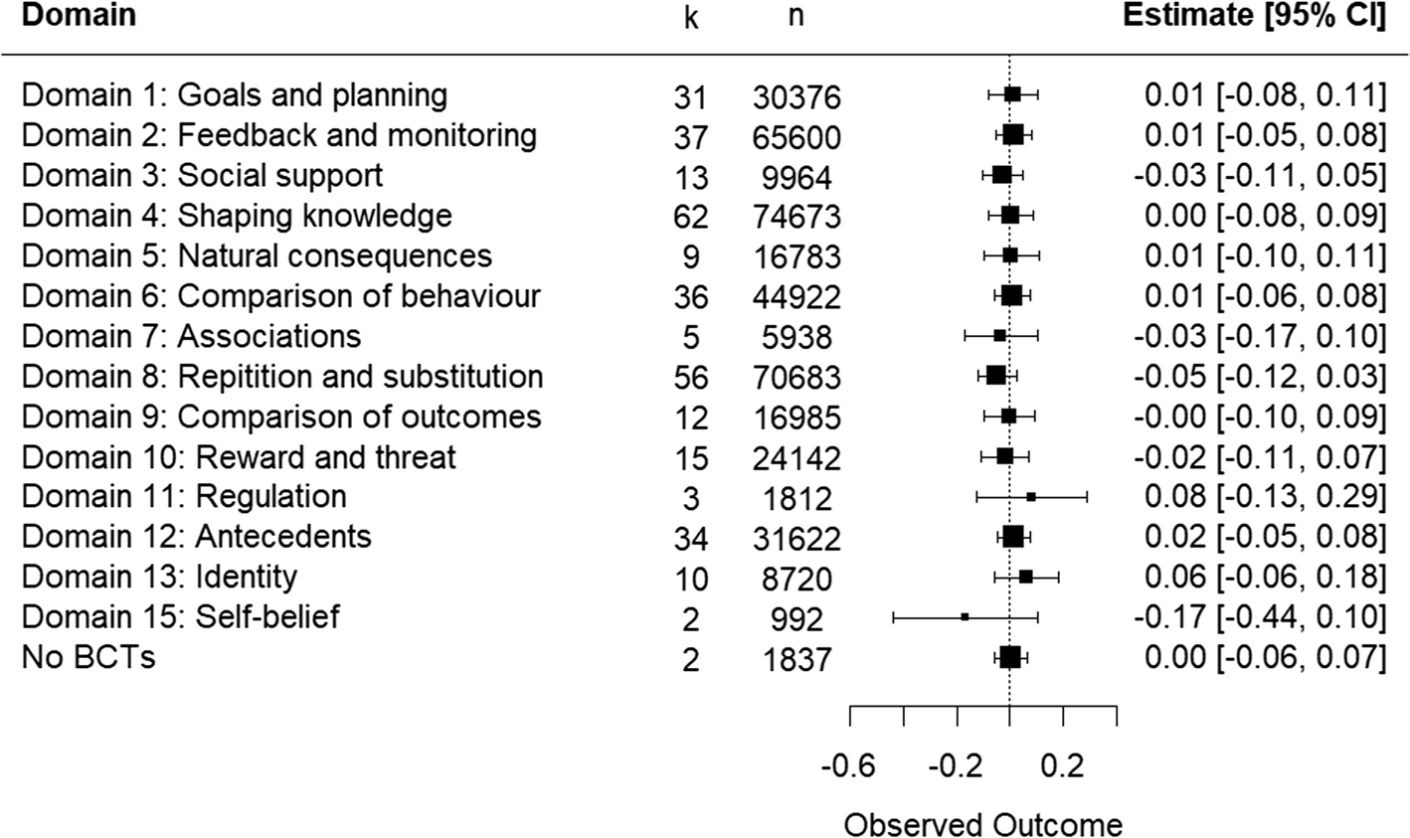
Meta-regression showing effects of BCT domains in physical activity interventions on child weight

Meta-regression of 78 PA intervention components (n = 97,517 participants) comparing BCT domains revealed none of the domains had a statistically significant association on child weight (Fig. 3) and a test of moderator difference showed no differences in effect between domains (Q_m_ = 0.59, I^2^ = 13%; Fig. 3). The heterogeneity was low (I^2^ = 19.95%, Tau^2^ = 0) [44]. However, the CI for *I*^2^ was quite wide, and it may vary from 9.84% to 69.69%.

### Aim 2b: Meta-regression analysis of individual BCTs and child weight

#### Healthy eating

Meta-regression of 67 HE intervention components (n = 92,256 participants) comparing individual BCTs revealed none of the individual BCTs had a statistically significant association on child weight (Supplementary file 4), and a test of moderator differences showed no difference in effect between the BCTs (Q_m_ = 0.5). The heterogeneity was high (I^2^ = 68.77%, Tau^2^ = 0.01) [44].

#### Physical activity

Meta-regression of 78 PA intervention components (n = 97,517 participants) comparing individual BCTs revealed none of the individual BCTs had a statistically significant association on child weight (Supplementary file 5) and a test of moderator differences found no difference in effect between the BCTs (Q_m_ = 0.57). The heterogeneity was low (I^2^ = 28.58%, Tau^2^ = 0.003) [44]. However, the CI for *I*^2^ was quite wide, and it may vary from 9.84% to 67.26%.

### Aim 2c: Discrete individual BCTs or combinations of BCTs.

The majority of trials used discrete individual BCTs or combination of BCTs in interventions. Supplementary file 6 and 7 shows the frequency of how distinct BCTs were grouped in HE and PA intervention components, where the frequency was more than one trial.

Within the HE intervention components, the most commonly used discrete individual BCTs or combination of BCTs was BCT *Instruction on how to perform the behaviour* alone (5 trials; Supplementary file 6). Within the PA intervention components, the most common discrete individual BCTs or combination of BCTs were BCTs *Instruction on how to perform the behaviour*, *Behavioural practice/rehearsal,* and *Demonstration of the behaviour* (5 trials; Supplementary file 7).

### Aim 2c: Meta-regression of discrete individual BCTs or combination of BCTs and child weight

#### Healthy eating

Meta-regression of discrete individual BCTs or combinations of BCTs included in HE intervention components found that none of the BCTs had any statistically significant association on child weight (Table 4), and a test of moderator differences failed to show a statistically significant difference in the effect of the combinations (Q_m_ = 7.19). The heterogeneity was moderate (I^2^ = 47.47%, Tau^2^ = 0.01).

**Table 4:** Meta-regression showing effects of discrete individual/combinations of BCT in HE interventions on child weight

BCTs	SMD	95% CI	Number of participants	Number of trials
4.1	−0.132	−0.268, 0.003	2,938	5
4.1, 8.1, 12.5, 6.1, 12.1	−0.007	−0.149, 0.134	3,236	3
4.1, 12.5	−0.023	−0.163, 0.117	2,239	3
4.1, 12.5, 12.1, 9.1	−0.001	−0.192, 0.189	1,332	2
4.1, 8.1, 12.1	0.036	−0.213, 0.285	1,158	2
4.1, 8.1, 12.5, 6.1	−0.037	−0.247, 0.172	7,735	2
4.1, 12.5, 12.1	−0.017	−0.371, 0.338	6,366	2
4.1, 12.5, 5.1, 2.2, 6.2	0.241	−0.043, 0.525	2,292	2

Tau^2^ = 0.01 (95% CI: 0, 0.04); I^2^ = 47.47 (95% CI: 0, 80.94)

Test for subgroup differences: Q = 6.16, df = 7, *p* = 0.522

BCT 2.2 Feedback on behaviour; 4.1 Instructions on how to perform the behaviour; 5.1 Information about health consequences; 6.1 Demonstration of the behaviour; 6.2 Social comparison; 8.1 Behavioural practice/rehearsal; 9.1 Credible source; 12.1 Restructuring the physical environment; 12.5 Adding objects to the environment

### Physical activity

Meta-regression of discrete individual BCTs or combinations of BCTs included in PA intervention components found that none of the BCTs had any statistically significant association on child weight (Table 5), and a test of moderator differences failed to show a statistically significant difference in the effect of the combinations (Q_m_ = 7.01). The heterogeneity was moderate to high (I^2^ = 57.76%, Tau^2^ = 0.02).

**Table 5:** Meta-regression showing effects of discrete individual/combinations of BCT in PA interventions on child weight

BCTs	SMD	95% CI	Number of participants	Number of trials
4.1, 8.1, 6.1	0.007	−0.142, 0.155	3,200	5
8.1	0.186	−0.094, 0.465	964	3
4.1, 8.1	−0.028	−0.344, 0.287	4,714	2
4.1	−0.317	−0.645, 0.011	511	2
4.1, 8.1, 6.1, 2.5	−0.001	−0.295, 0.293	7,712	2
12.5	0.049	−0.307, 0.405	474	2

Tau^2^ = 0.02 (95% CI: 0, 0.17); I^2^ = 57.76% (95% CI: 11.8, 92.69)

Test for subgroup differences: Q = 7.01, df = 5, *p* = 0.22

BCT 2.5 Monitoring of outcome(s) of behaviour without feedback; 4.1 Instructions on how to perform the behaviour; 6.1 Demonstration of the behaviour; 8.1 Behavioural practice/rehearsal; 12.5 Adding objects to the environment

### Meta-CART

When applying meta-CART to both HE and PA interventions, with all BCTs included as moderators, no moderator effect was found, despite liberal growing and pruning parameters (minimum node size of two, decrease in between trial heterogeneity of 0 and pruning parameter of 0).

## Discussion

As far as we know, this study is the first to explore the associations between BCTs and the impact on BMI/BMI z-scores of school-based obesity prevention interventions supporting children aged 6–18 years. The study found HE interventions adopting three particular BCT domains were significantly associated with a positive effect on child weight. Whereas no significant associations were found between PA interventions adopting any BCT domain and child weight, or for individual BCTs, discrete individual BCTs or combinations of BCTs and meta-CART analysis for either HE or PA interventions.

Of the 55 unique BCTs identified across school-based childhood obesity prevention interventions, the five most common were *Instruction on how to perform the behaviour*, *Behavioural practice/rehearsal*, *Restructuring the physical environment* (HE only), *Adding objects to the environment*, *Demonstration of the behaviour* and *Self-monitoring of behaviour* (PA only). These findings are consistent with a previous scoping review that explored the use of BCT taxonomies in childhood obesity prevention interventions supporting children 12 years or younger in 54 different trials [27]. Our study identified several BCT domains and individual BCTs that were not used in childhood obesity prevention intervention. A total of two BCT domains (*Scheduled consequences* and *Covert learning*) and 38 unique BCTs were not coded in this study. It is possible that these were not used due to lack of suitability for the target population (i.e. children), such as BCTs that involves *punishments*, *behavioural experiments* or delivery context (including inappropriate for delivery in the schools setting); or are not appropriate for the intervention target (obesity prevention) context such as *social incentives*, *body changes* and *satiation;* or difficult to implement in a RCT, such as *reduce reward frequency*, but this was not examined in the study. However, there are other unutilised BCTs that are an opportunity for new interventions. Current literature does not have a recommendation on these unutilised BCTs, probably because of infrequent usage in the first place but BCTs such *as identity associated with changed behaviour* and *mental rehearsal of successful performance* could be used to improve students’ self-esteem to eat healthier or increase physical activity. The complexity of interventions such as those that uses Health Promoting Schools framework [47], could also have an impact on the types of BCTs used by schools and is something further research could look to investigate. For example, other reviews have investigated the influence other characteristics may have on child weight, such as who delivered the intervention [15], however this was outside the scope of our study.

Findings from the discrete individual BCTs or combinations of BCTs (Supplementary file 6 and 7) showed that HE intervention components commonly used *Instruction on how to perform the behaviour* alone to change behaviour. Studies have found that a lack nutritional knowledge by students and parents has been a common barrier and has recommended addressing it in school-based obesity prevention interventions [48, 49] and thus, it was not surprising that this BCT was frequently used. However, research has also suggested that nutrition knowledge alone is insufficient to result in a change of behaviour [50], and this could explain why despite *Instruction on how to perform the behaviour* is frequently used, but was not found to be associated with reduced BMI/BMI z-scores. Within PA intervention components however, the most used combination of BCTs were *Instruction on how to perform the behaviour, Demonstration of the behaviour* and *Behavioural practice/rehearsal*. In addition to a lack of knowledge, a lack of skills and beliefs in one’s capabilities are common barriers to PA among adolescents [51], which are likely best addressed by such BCTs. It is reassuring that the most commonly applied BCTs are ones that target the most frequently report barriers to HE and PA. Efforts to map specific BCTs that may target barriers to changing behaviour is in progress by the Theory & Techniques Tool [19] and these provide significant opportunity to better optimise the impact of obesity prevention interventions.

Findings from the meta-regressions within HE intervention components showed that the following BCT domains, *Goals and planning, Social support* and *Comparison of behaviour* were significantly associated with a positive effect on child weight. The most frequently coded BCTs within their domains were *Problem solving* (n = 15, 27%)*, Goal setting (outcome)* (n = 15, 27%)*, Social support (unspecified)* (n = 11, 92%) *and Demonstration of the behaviour* (n = 28, 82%). This finding is supported by other studies in obesity prevention interventions [22, 30]. For example, Ashton et al. (2020) found BCTs *Goal-setting (outcome), Self-monitoring of behaviour*, and *Social support (unspecified)* were effective BCTs influencing weight outcomes in young adults (17–35 years) [30], and Dombrowski et al. (2010), using the previous BCT taxonomy (Abraham and Michie 2008 [52]), found BCTs *provision of instructions, self-monitoring, relapse prevention* and *prompting practice* were linked to more successful interventions in adults with obesity [22]*.* In the current study, BCTs in domain 6 were also associated with reduced BMI/BMI z-scores, which was not the case in the above two studies. This could be explained by the variations in methodology such as BCT taxonomy used (Abraham and Michie 2008 [52]), study population (post-partum women, young adults, adults with obesity) and method of BCT analysis (percentage effectiveness ratio). Furthermore, whilst schools are a recommended setting, the primary purpose of schools is to educate children and therefore may be organised in ways that hinders obesity prevention interventions [53].

For PA intervention components, our study did not find any BCTs that were significantly associated with child weight. This was in contrast to other another review which found that BCTs *goal setting* (SMD 0.29, 95% CI 0.05, 0.53) *and feedback* (SMD 0.35, 95% CI 0.11, 0.60) were significantly associated with increased physical activity [54]. This other review examined impact on PA outcomes and only included hospitalised patients; differences in findings with our study could be due to variation in methodology, study population (hospitalised patients), BCT taxonomy used (CALO-RE) [55] and target outcome (increase PA). Further research is needed to confirm if any associations exist.

Despite the lack of statistically significant results within individual BCTs, there were nine BCTs within HE intervention components (*Goal setting (outcome), Restructuring the physical environment, Behavioural practice/rehearsal, Feedback on behaviour, Problem solving, Demonstration of the behaviour, Credible source, Adding objects to the environment* and *Information about health consequences)* and one BCT within PA intervention components (*Behavioural practice/rehearsal)* that had an effect size larger than the SMD of the previous review [16] (range from −0.05 to – 0.18) and a direction of effect in favour of obesity prevention. While this must be interpreted with caution given the low-certainty evidence, these findings provide some guidance regarding which BCTs could be prioritised for HE and PA intervention components of obesity prevention interventions.

The ability to identify effective individual BCTs was limited due to small overall effect size, despite three BCT domains having a significant association with a positive effect on child weight. However, these BCTs along with BCTs in beneficial direction provides the best evidence (as of now) until more studies and research are conducted. It is also worth noting that there were no associations for some of the most commonly used BCTs (Table 2 and 3), such as BCTs *Instruction on how to perform the behaviour* in HE intervention components, and BCTs *Self-monitoring of behaviour, Instructions on how to perform the behaviour, Demonstration of the behaviour and Adding objects to the environment* in PA intervention components, and may benefit from further investigations in the future.

Findings from the meta-regression analysis of discrete individual BCTs or combinations of BCTs, found no significant association with weight. This was not surprising as no individual BCTs were found to be significant and the use of meta regression relies on a large number of studies reporting the same combinations of BCTs to be able to detect the effects [56]. While our study had a large number of studies, it only had a small number of discretely used individual BCTs or combinations of BCTs. In our study, we looked at combinations of BCTs and undertook an exploratory meta-CART analysis which found no synergistic effects between BCTs and was limited by small sample sizes. Exploring the impact of common combinations of BCTs is likely to provide better insight into how interventions work as a whole, as few interventions consists of only one BCT. However, as our study demonstrate, current evaluation approaches prove challenging due to small sample sizes and the lack of specification of BCTs [56]. As the evidence grows, future research should report BCTs in a standardised manner using the emerging Behaviour Change Technique Ontology (BCTO) [57], consider sharing of intervention materials to allow for better classification and secondary data synthesis efforts similar to the meta-CART approach to better understand the specific components associated with effects of such interventions.

This study has several strengths that contributes to the literature. First, this study is the first international study to examine the effectiveness of BCTs in school-based obesity prevention interventions. This study also distinguishes itself through rigorous methodology such as using a comprehensive review to provide data for this study and for prospectively registering the study with PROSPERO (CRD42022366743). Besides that, this study used a range of analyses methods and synthesised combinations of BCTs, which is not commonly done. Lastly, being a secondary data analysis, this study repurposed data included in a high-quality systematic review which is recommended to reduce research waste, identify research gaps, and address important public health issues [58].

However, the study had some limitations. First, the coding was completed mostly by one coder (k = 95 trials) which was a change to the protocol, due to practical limitations. However, prior to single coding, both coders complete consolidation on the first 20% of trials and achieve reasonable agreement (81%). Second, the coding of BCTs was dependent on the description of intervention details reported by authors and has resulted in some trials reporting zero BCTs due to the lack of information (2.4%, n = 3). These limitations were minimised by reviewing and coding all available material provided by authors (e.g. protocols, additional files, and associate papers). We made a pragmatic decision to not seek unpublished documents based on the number of included trials (k = 124) and only 34% of authors in the field typically reply [59]. Furthermore, even if authors were contacted, the details were still lacking [60] and is a common practice among BCT studies [30, 61] but should be done if possible. Third, since undertaking this study, a new version of the taxonomy, the BCTO, has been published which future research should explore. Fourth, heterogeneity was high for the analysis of BCT domains and BMI/BMI z-scores in HE intervention components and could be investigated in future analysis such as with a qualitative comparative analysis. Fifth, this study only included RCTs and whilst other study designs such as non-randomised study designs could be more appropriate to account for the complexity of school-based interventions [62], they were not synthesised. Sixth, despite the use of meta-regression which is often considered the top of the evidence hierarchy, this study only takes a cross-section of a particular period (up to June 2021) and thus the findings cannot infer causation and only potential associations but provides the best evidence until further research is conducted. Lastly, the search for this study was conducted up to 30th June 2021, and while it is likely additional eligible trials have been published since, it is unlikely their inclusion would substantially alter the results, given the large number of trials already included and the small effect size observed (SMD −0.03, 95% CI −0.06, −0.01; 93 trials, n = 131,443). Nevertheless, future research should aim to update the search to reflect more recent evidence, especially considering the potential increase in use of BCTs, be dual coded and attempt to seek unpublished documents, if possible, to reduce potential systematic and random errors.

## Conclusion

This study found BCTs in domains *Shaping knowledge* (e.g. *Instructions on how to perform the behaviour*) and *Repetition and substitution* (e.g. *Behavioural practice/rehearsal*) are most frequently used in HE and PA intervention components respectively, while BCTs in domains *Goals and planning* (e.g. *Goal setting (outcome)*)*, Social support* (e.g. *social support (unspecified)*) and *Comparison of behaviour* (e.g. *Demonstration of the behaviour*) of HE intervention components were associated with a positive effect on child weight and should be prioritised in future school-based obesity prevention interventions. BCTs *Instruction on how to perform the behaviour* and *Behavioural practice/rehearsal* were the most frequently used BCTs in both HE and PA intervention components however these were not associated with child weight. Meta-regression of discrete individual BCTs or combinations of BCTs and meta-CART analysis both had insufficient trials to find any associations. While this research identified some BCTs which could be prioritised for future HE interventions, little is known about the BCTs for PA interventions and the combination of BCTs that may be most effective.

## Supplementary Information

Supplementary file 1.

Supplementary file 2.

Supplementary file 3.

Supplementary file 4.

Supplementary file 5.

Supplementary file 6.

Supplementary file 7.

## Data Availability

Data is provided within the manuscript or supplementary information files. The datasets used and/or analysed during the current study are available from the corresponding author on reasonable request.

## Abbreviations

BCTO: Behaviour Change Technique Ontology
BCTs: Behaviour Change Techniques
BCTTv1: Behaviour Change Technique Taxonomy v1
BMI: Body Mass Index
BMI z-score: Body Mass Index z-score
CALO-RE: Coventry, Aberdeen and London-Refined
CART: Classification and Regression Tree
CENTRAL: Cochrane Central Register of Controlled Trials
CI: Confidence Interval
CINAHL: Cumulative Index to Nursing and Allied Health Literature
HE: Health Eating
NHMRC: National Health and Medical Research Council
PA: Physical Activity
PROSPERO: International Prospective Register of Systematic Reviews
RCTs: Randomised Controlled Trials
REDCap: Research Electronic Data Capture
SMD: Standardised Mean Difference
WHO: World Health Organisation
