# Supplementary material for: Identifying behaviour change techniques in school-based childhood obesity prevention interventions: a secondary analysis of a systematic review
Source: BMC Public Health. 2025 Jul 2;25:2250. doi: 10.1186/s12889-025-23421-9 (PMC12219750; doi:10.1186/s12889-025-23421-9)
Supplement: Supplementary file 1 [file 12889_2025_23421_MOESM1_ESM.docx]

**BCT Codebook**

**Target population:** Students, parents, teachers

**Target behaviours:** Healthy eating or Physical activity

**Target outcomes:** BMI or BMI z-scores

| **No.** | **Label** | **Definition** | **Coding tips** |
| --- | --- | --- | --- |
| General tips and rules | | We will code a BCT if the author of the paper reports BCTs using the BCTTv1 or older versions (e.g. Abraham and Mitchie 2008) | |
| **1.1** | Goal setting (behaviour) | Set or agree on a goal defined in terms of the behavior to be achieved.  *Note: only code goal-setting if there is sufficient evidence that goal set as part of intervention;* *if goal unspecified or a behavioral outcome, code* ***1.3, Goal setting (outcome)****; if the goal defines a specific context, frequency, duration or intensity for the behavior, also code* ***1.4, Action planning*** | - Code if study reports a specific goal that is a behaviour |
| **1.3** | Goal setting (outcome) | Set or agree on a goal defined in terms of a positive **outcome** of wanted behavior.  *Note:* *only code guidelines if set as a goal in an intervention context; if goal is a behavior, code* ***1.1, Goal setting (behavior)****; if goal unspecified code* ***1.3, Goal setting (outcome)*** | - Code if study reports goal setting but unspecific - E.g. Participants will be provided with pedometers and **encouraged to initiate goal setting** and self-monitoring behaviours. |
| **2.1** | Monitoring of behaviour by others without feedback | Observe or record behavior with the person’s knowledge as part of a behavior change strategy.  *Note: if monitoring is part of a data collection procedure rather than a strategy aimed at changing behavior, do not code; if feedback given, code only* ***2.2, Feedback on behavior****, and not* ***2.1, Monitoring of behavior by others without feedback****; if monitoring outcome(s) code* ***2.5, Monitoring outcome(s) of behavior by others without feedback****; if self-monitoring behavior, code* ***2.3, Self-monitoring of behaviour*** | - Keep a look out as this BCT is uncommon but possible |
| **4.1** | Instructions on how to perform the behaviour | Advise or agree on how to perform the behavior (includes ‘**Skills training**’) *Note: when the person attends classes such as exercise or cookery, code* ***4.1, Instruction on how to perform the behavior, 8.1, Behavioral practice/rehearsal*** *and* ***6.1, Demonstration of the behavior*** | - Providing flyers is not sufficient to code due to it being considered a passive intervention and we cannot be certain they received it. (e.g. children given flyers to give parents). - Includes provision of knowledge - Includes workshops |
| **4.2** | Information about antecedents | Provide information about antecedents (*e.g. social and environmental situations and events, emotions, cognitions)* that reliably predict performance of the behaviour | - Keep a look out as this BCT is uncommon but possible |
| **5.1** | Information about health consequences | Provide information (e.g. written, verbal, visual) about health consequences of performing the behavior.  *Note: consequences can be for any target, not just the recipient(s) of the intervention; emphasising importance of consequences is not sufficient; if information about emotional consequences, code* ***5.6, Information about emotional consequences****; if about social, environmental or unspecified consequences code* ***5.3,*** ***Information about social and environmental consequences*** | - Only code if consequences is provided |
| **6.1** | Demonstration of the behavior | Provide an observable sample of the performance of the behaviour, directly in person or indirectly e.g. via film, pictures, for the person to aspire to or imitate (includes ‘**Modelling**’).  *Note:* if advised to practice, also code, ***8.1, Behavioural practice and rehearsal;*** *If provided with instructions on how to perform, also code* ***4.1, Instruction on how to perform the behaviour*** | - Includes workshops |
| **7.1** | Prompts/cues | Introduce or define environmental or social stimulus with the purpose of prompting or cueing the behavior. The prompt or cue would normally occur at the time or place of performance *Note: when a stimulus is linked to a specific action in an if-then plan including one or more of frequency, duration or intensity* *also code* ***1.4, Action planning****.* | - Providing flyers is insufficient to code due to it being considered a passive intervention and we cannot be certain they received it. (e.g. children given flyers to give parents). - Putting up posters is insufficient to code, unless at a location that prompts the behavior. For example at point of sales |
| **8.1** | Behavioral practice/ rehearsal | Prompt practice or rehearsal of the performance of the behavior one or more times in a context or at a time when the performance may not be necessary, in order to increase habit and skill.  *Note: if aiming to associate performance with the context, also code* ***8.3, Habit formation*** | - Includes workshops |
| **8.2** | Behavior substitution | Prompt substitution of the unwanted behavior with a wanted or neutral behavior  *Note: if this occurs regularly, also code* ***8.4, Habit reversal*** | - E.g. Swapping unhealthy food choices for healthier choices |
| **8.3** | Habit formation | Prompt rehearsal and repetition of the behavior in the same context repeatedly so that the context elicits the behavior *Note: also code* ***8.1, Behavioral practice/rehearsal*** | - i.e. Repeating the behaviour consistently |
| **8.6** | Generalisation of target behaviour | Advise to perform the wanted behaviour, which is already performed in a particular situation, in another situation | - Keep a look out as this BCT is uncommon but possible |
| **8.7** | Graded tasks | Set easy-to-perform tasks, making them increasingly difficult, but achievable, until behavior is performed | - Keep a look out as this BCT is uncommon but possible |
| **9.2** | Pros and cons | Advise the person to identify and compare reasons for wanting (pros) and not wanting to (cons) change the behavior (includes ‘**Decisional balance’***)* *Note:* *if providing information about health consequences, also code* ***5.1, Information about health consequences****; if providing information about emotional consequences, also code* ***5.6, Information about emotional consequences****; if providing information about social, environmental or unspecified* | - Keep a look out as this BCT is uncommon but possible |
| **10.1** | Material incentive (behavior) | Inform that money, vouchers or other valued objects ***will be*** delivered if and only if there has been effort and/or progress in performing the behavior (includes ***‘*Positive reinforcement’**).  *Note: if incentive is social, code* ***10.5, Social incentive*** *if unspecified code* ***10.6,*** ***Non-specific incentive,*** *and not* ***10.1, Material incentive (behavior)****; if incentive is for* ***outcome,*** *code* ***10.8, Incentive (outcome).*** *If reward is delivered also code one of:* ***10.2, Material reward (behavior); 10.3, Non-specific reward; 10.4, Social reward, 10.9, Self-reward; 10.10, Reward (outcome)*** | - Only code if it is an incentive and a reward was not given - E.g. The student enters a raffle if he/she performs the behavior |
| **10.2** | Material reward (behavior) | Arrange for the delivery of money, vouchers or other valued objects if and only if there ***has been*** effort and/or progress in performing the behavior (includes ‘**Positive reinforcement’**) *Note: If reward is social, code* ***10.4, Social reward****, if unspecified code* ***10.3, Nonspecific reward****, and not* ***10.1, Material reward (behavior)****; if reward is for* ***outcome****, code* ***10.10, Reward (outcome).*** *If informed of reward in advance of rewarded behaviour, also code one of:* ***10.1, Material incentive (behaviour); 10.5, Social incentive; 10.6, Non-specific incentive; 10.7, Self-incentive; 10.8, Incentive (outcome)*** | - Only code if a reward is given - E.g. The student is given a water bottle if he/she performs the behavior |
| **10.10** | Reward (outcome) | Arrange for the delivery of a reward if and only if there ***has been*** effort and/or progress in achieving the behavioral **outcome** (includes ‘**Positive reinforcement**’).  *Note: this includes social, material, self- and non-specific rewards for outcome; if reward is for the* ***behavior*** *code* ***10.4****,* ***Social*** ***reward****,* ***10.2, Material*** ***reward (behavior)****,* ***10.3,*** ***Non****-****specific*** ***reward*** *or* ***10.9****,* ***Self****-****reward*** *and not* ***10.10, Reward (outcome).*** *If informed of reward in advance of rewarded behaviour, also code one of****: 10.1, Material incentive (behaviour); 10.5, Social incentive; 10.6,***  ***Non-specific incentive; 10.7, Self-incentive; 10.8, Incentive (outcome)*** | - Only code if providing a reward for achieving the outcome |
| **12.1** | Restructuring the physical environment | Change, or advise to change the **physical** environment in order to facilitate performance of the wanted behavior or create barriers to the unwanted behavior (other than prompts/cues, rewards and punishments).  *Note: this may also involve* ***12.3, Avoidance/reducing exposure to cues for the behavior****;* *if restructuring of the social environment code* ***12.2, Restructuring the social environment;*** *if only adding objects to the environment, code* ***12.5, Adding objects to the environment*** | - Only code if there is a change to the physical environment, rather than adding a new object - E.g. Taking out PA equipment during lunch break |
| **12.5** | Adding objects to the environment | Add objects to the environment in order to facilitate performance of the behavior.  *Note: Provision of information (e.g. written, verbal, visual) in a booklet or leaflet is insufficient. If this is accompanied by social support, also code* ***3.2, Social support (practical)****; if the environment is changed beyond the addition of objects, also code* ***12.1, Restructuring the physical environment*** | - Putting up posters is insufficient to code, unless it is a core component of the intervention such as placing posters at point of sales to prompts the behavior. - Only code if there is an addition to the environment - E.g. Adding new PA equipment during lunch break |
