# Supplementary material for: Identifying behaviour change techniques in school-based childhood obesity prevention interventions: a secondary analysis of a systematic review
Source: BMC Public Health. 2025 Jul 2;25:2250. doi: 10.1186/s12889-025-23421-9 (PMC12219750; doi:10.1186/s12889-025-23421-9)
Supplement: Supplementary file 2 [file 12889_2025_23421_MOESM2_ESM.docx]

**List of included studies** (n = 124)

| **Study ID** | **Included in meta-analysis?** | **Reference** |
| --- | --- | --- |
| Adab 2018 | Yes | Adab P, Pallan MJ, Lancashire ER, Hemming K, Frew E, Barrett T, et al. Effectiveness of a childhood obesity prevention programme delivered through schools, targeting 6 and 7 year olds: cluster randomised controlled trial (WAVES study). BMJ. 2018;360:k211. |
| Amaro 2006 | Yes | Amaro SV, A. Di Costanzo, A. Madeo, I. Viggiano, A. Baccari, M. E. et, a l. Kalèdo, a new educational board-game, gives nutritional rudiments and encourages healthy eating in children: a pilot cluster randomized trial. European Journal of Pediatrics. 2006;165(9):630-5. |
| Andrade 2014 | Yes | Andrade SL, C. Ochoa-Aviles, A. Verstraeten, R. Huybregts, L. Roberfroid, D. Andrade, D. Camp, J. V. Rojas, R. Donoso, S. Cardon, G. Kolsteren, P. A school-based intervention improves physical fitness in Ecuadorian adolescents: a cluster-randomized controlled trial. Int. 2014;11:153. |
| Arlinghaus 2021 | No | Arlinghaus KR, Ledoux TA, Johnston CA. Randomized Controlled Trial to Increase Physical Activity Among Hispanic-American Middle School Students. The Journal of school health. 2021;02. |
| Bogart 2016 | No | Bogart LME, M. N. Cowgill, B. O. Klein, D. J. Hawes-Dawson, J. Uyeda, K. Schuster, M. A. Two-Year BMI Outcomes From a School-Based Intervention for Nutrition and Exercise: A Randomized Trial. Pediatrics. 2016;137(5). |
| Bonsergent 2013 | Yes | Bonsergent EA, N. Thilly, N. Tessier, S. Legrand, K. Lecomte, E. Aptel, E. Hercberg, S. Collin, J. F. Briançon, S. Pralimap Trial, G. r o u p. Overweight and obesity prevention for adolescents: A cluster randomized controlled trial in a school setting. Am J Prev Med. 2013;44(1):30-9. |
| Brandstetter 2012 | Yes | Brandstetter SK, J. Berg, S. Galm, C. Fritz, M. Peter, R. Prokopchuk, D. Steiner, R. Overweight prevention implemented by primary school teachers: a randomised controlled trial. Obesity Facts. 2012;5(1):1-11. |
| Breheny 2020 | Yes | Breheny K, Passmore S, Adab P, Martin J, Hemming K, Lancashire ER, et al. Effectiveness and cost-effectiveness of The Daily Mile on childhood weight outcomes and wellbeing: a cluster randomised controlled trial. International journal of obesity. 2020. |
| Brito Beck da Silva 2019 | Yes | Brito Beck da Silva K, Ortelan N, Giardini Murta S, Sartori I, Couto RD, Leovigildo Fiaccone R, et al. Evaluation of the Computer-Based Intervention Program Stayingfit Brazil to Promote Healthy Eating Habits: The Results from a School Cluster-Randomized Controlled Trial. Int J Environ Res Public Health. 2019;16(10):14. |
| Caballero 2003 | Yes | Caballero BC, T. Davis, S. M. Ethelbah, B. Rock, B. H. Lohman, T. et, a l. Pathways: a school-based, randomized controlled trial for the prevention of obesity in American Indian schoolchildren. American Journal of Clinical Nutrition. 2003;78(5):1030-8. |
| Cao 2015 | Yes | Cao ZJW, S. M. Chen, Y. A randomized trial of multiple interventions for childhood obesity in China. Am J Prev Med. 2015;48(5):552-60. |
| Carlin 2018 | No | Carlin A, Murphy MH, Nevill A, Gallagher AM. Effects of a peer-led Walking In ScHools intervention (the WISH study) on physical activity levels of adolescent girls: a cluster randomised pilot study. Trials [Electronic Resource]. 2018;19(1):31. |
| Christiansen 2013 | No | Christiansen LBT, M. Boyle, E. Kristensen, P. L. Troelsen, J. Effect of a school environment intervention on adolescent adiposity and physical fitness. Scandinavian Journal of Medicine & Science in Sports. 2013;23(6):e381-e9. |
| Clemes 2020 | Yes | Clemes SA, Bingham DD, Pearson N, Chen Y-L, Edwardson CL, McEachan RRC, et al. Stand Out in Class: restructuring the classroom environment to reduce sitting time – findings from a pilot cluster randomised controlled trial. International Journal of Behavioral Nutrition and Physical Activity. 2020;17(1):55. |
| Coleman 2012 | No | Coleman KJS, M. Caparosa, S. L. Pomichowski, M. E. Dzewaltowski, D. A. The healthy options for nutrition environments in schools (Healthy ONES) group randomized trial: using implementation models to change nutrition policy and environments in low income schools. Int. 2012;9:80-. |
| Cunha 2013 | No | Cunha DBdSB, S. Pereira, R. A. Sichieri, R. Effectiveness of a randomized school-based intervention involving families and teachers to prevent excessive weight gain among adolescents in Brazil. PLoS ONE. 2013;8(2):e57498-e. |
| Damsgaard 2014 | Yes | Damsgaard CTD, S. M. Laursen, R. P. Ritz, C. Hjorth, M. F. Lauritzen, L. Sørensen, L. B. Petersen, R. A. Andersen, M. R. Stender, S. Andersen, R. Tetens, I. Mølgaard, C. Astrup, A. Michaelsen, K. F. Provision of healthy school meals does not affect the metabolic syndrome score in 8-11-year-old children, but reduces cardiometabolic risk markers despite increasing waist circumference. Br J Nutr. 2014;112(11):1826-36. |
| Davis 2021 | Yes | Davis JN, Pérez A, Asigbee FM, Landry MJ, Vandyousefi S, Ghaddar R, et al. School-based gardening, cooking and nutrition intervention increased vegetable intake but did not reduce BMI: Texas sprouts - a cluster randomized controlled trial. Int. 2021;18(1):1-14. |
| de Greeff 2016 | Yes | de Greeff JWH, E. Mullender-Wijnsma, M. J. Bosker, R. J. Doolaard, S. Visscher, C. Effect of Physically Active Academic Lessons on Body Mass Index and Physical Fitness in Primary School Children. Journal of School Health. 2016;86(5):346-52. |
| Dewar 2013 | Yes | Dewar DLM, P. J. Plotnikoff, R. C. Okely, A. D. Collins, C. E. Batterham, M. Callister, R. Lubans, D. R. The nutrition and enjoyable activity for teen girls study: a cluster randomized controlled trial. Am J Prev Med. 2013;45(3):313-7. |
| Donnelly 2009 | Yes | Donnelly JEG, J. L. Gibson, C. A. Smith, B. K. Washburn, R. A. Sullivan, D. K. et, a l. Physical Activity Across the Curriculum (PAAC): a randomized controlled trial to promote physical activity and diminish overweight and obesity in elementary school children. Prev Med2009. p. 336-41. |
| Drummy 2016 | Yes | Drummy CM, E. M. McKee, D. P. Breslin, G. Davison, G. W. Murphy, M. H. The effect of a classroom activity break on physical activity levels and adiposity in primary school children. Journal of Paediatrics and Child Health. 2016. |
| Duncan 2019 | Yes | Duncan S, Stewart T, McPhee J, Borotkanics R, Prendergast K, Zinn C, et al. Efficacy of a compulsory homework programme for increasing physical activity and improving nutrition in children: a cluster randomised controlled trial. Int. 2019;16(1):80. |
| Ezendam 2012 | Yes | Ezendam NPB, J. Oenema, A. Evaluation of the Web-based computer-tailored FATaintPHAT intervention to promote energy balance among adolescents: results from a school cluster randomized trial. Archives of pediatrics & adolescent medicine. 2012;166(3):248-55. |
| Fairclough 2013 | Yes | Fairclough SJH, A. F. Davies, I. G. Gobbi, R. Mackintosh, K. A. Warburton, G. L. Stratton, G. van Sluijs, E. M. Boddy, L. M. Promoting healthy weight in primary school children through physical activity and nutrition education: a pragmatic evaluation of the CHANGE! randomised intervention study. BMC Public Health. 2013;13:626-. |
| Farias 2015 | No | Farias Edos SG, E. M. Morcillo, A. M. Guerra-Junior, G. Amancio, O. M. Effects of programmed physical activity on body composition in post-pubertal schoolchildren. J Pediatr (Rio J). 2015;91(2):122-9. |
| Farmer 2017 | Yes | Farmer VLW, S. M. Mann, J. I. Schofield, G. McPhee, J. C. Taylor, R. W. The effect of increasing risk and challenge in the school playground on physical activity and weight in children: a cluster randomised controlled trial (PLAY). International Journal of Obesity. 2017;41(5):793-800. |
| Ford 2013 | Yes | Ford PA, Perkins G, Swaine I. Effects of a 15-week accumulated brisk walking programme on the body composition of primary school children. Journal of Sports Sciences. 2013;31(2):114-22. |
| Foster 2008 | Yes | Foster GDS, S. Borradaile, K. E. Grundy, K. M. Vander Veur, S. S. Nachmani, J. et, a l. A policy-based school intervention to prevent overweight and obesity. Pediatrics2008. p. e794-e802. |
| Gentile 2009 | Yes | Gentile DAW, G. Eisenmann, J. C. Reimer, R. A. Walsh, D. A. Russell, D. W. et, a l. Evaluation of a multiple ecological level child obesity prevention program: Switch what you Do, View, and Chew. BMC Med2009. p. 49-. |
| Gortmaker 1999a | No | Gortmaker SLP, K. Wiecha, J. Sobal, A. M. Dixit, S. Fox, M. K. et, a l. Reducing obesity via a school-based interdisciplinary intervention among youth. Archives of Pediatrics and Adolescent Medicine. 1999;153(4):409-18. |
| Greve 2015 | Yes | Greve JH, E. Evaluating the impact of a school-based health intervention using a randomized field experiment. Econ Hum Biol. 2015;18:41-56. |
| Grydeland 2014 | Yes | Grydeland MB, M. Anderssen, S. A. Klepp, K. I. Bergh, I. H. Andersen, L. F. Ommundsen, Y. Lien, N. Effects of a 20-month cluster randomised controlled school-based intervention trial on BMI of school-aged boys and girls: the HEIA study. British Journal of Sports Medicine. 2014;48(9):768-73. |
| Habib-Mourad 2014 | Yes | Habib-Mourad C. An intervention to promote Healthy Eating and Physical Activity in Lebanese School children: Health-E-PALS, a pilot cluster randomised controlled trial. Obesity Facts. 2014;21th European Congress on Obesity (ECO2014):158-9. |
| Habib-Mourad 2020 | No | Habib-Mourad C, Ghandour LA, Maliha C, Dagher M, Kharroubi S, Hwalla N. Impact of a Three-Year Obesity Prevention Study on Healthy Behaviors and BMI among Lebanese Schoolchildren: Findings from Ajyal Salima Program. Nutrients. 2020;12(9). |
| Haerens 2006 | Yes | Haerens LD, B. Maes, L. Cardon, G. Stevens, V. De Bourdeaudhuij, I. Evaluation of a 2-year physical activity and healthy eating intervention in middle school children. Health Educ Res. 2006;21(6):911-21. |
| Han 2006 | No | Han XL, P. Chen, Y. The Outcome Evaluation of the Elementary Students about 3-year in System-intervention of Nutrition Dinner in Yangpu District, Shanghai. Health Education and Health Promotion. 2006;1:21-4. |
| Harrington 2018 | Yes | Harrington DM, Davies MJ, Bodicoat DH, Charles JM, Chudasama YV, Gorely T, et al. Effectiveness of the 'Girls Active' school-based physical activity programme: A cluster randomised controlled trial. Int. 2018;15(1):40. |
| HEALTHY Study Gp 2010 | Yes | Healthy Study Group. A school-based intervention for diabetes risk reduction. New England journal of medicine. 2010;363(5):443-53. |
| Hendy 2011 | No | Hendy HMW, K. E. Camise, T. S. Kid's Choice Program improves weight management behaviors and weight status in school children. Appetite. 2011;56(2):484-94. |
| Herscovici 2013 | Yes | Herscovici CRK, I. De Gregorio, M. J. Gender differences and a school-based obesity prevention program in Argentina: a randomized trial. Rev Panam Salud Publica. 2013;34(2):75-82. |
| Hollis 2016 | Yes | Hollis JLS, R. Campbell, L. Morgan, P. J. Lubans, D. R. Nathan, N. Wolfenden, L. Okely, A. D. Davies, L. Williams, A. Cohen, K. E. Oldmeadow, C. Gillham, K. Wiggers, J. Effects of a 'school-based' physical activity intervention on adiposity in adolescents from economically disadvantaged communities: secondary outcomes of the 'Physical Activity 4 Everyone' RCT. International Journal of Obesity. 2016;40(10):1486-93. |
| Ickovics 2019 | No | Ickovics JR, Duffany KO, Shebl FM, Peters SM, Read MA, Gilstad-Hayden KR, et al. Implementing School-Based Policies to Prevent Obesity: Cluster Randomized Trial. Am J Prev Med. 2019;56(1):e1-e11. |
| James 2004 | Yes | James JT, P. Cavan, D. Kerr, D. Preventing childhood obesity by reducing consumption of carbonated drinks: cluster randomised controlled trial. BMJ. 2004;328(7450):22-. |
| Jansen 2011 | Yes | Jansen WB, G. Meima, A. Zwanenburg, E. J. V. Mackenbach, J. Effectiveness of a primary school-based intervention to reduce overweight. International Journal of Pediatric Obesity. 2011;6(2-2):e70-e7. |
| Johnston 2013 | Yes | Johnston CAM, J. P. El-Mubasher, A. Gallagher, M. Tyler, C. Woehler, D. Impact of a school-based pediatric obesity prevention program facilitated by health professionals. Journal of School Health. 2013;83(3):171-81. |
| Kain 2014 | Yes | Kain JC, F. Moreno, L. Leyton, B. School-based obesity prevention intervention in Chilean children: effective in controlling, but not reducing obesity. J Obes. 2014;2014:618293-. |
| Kennedy 2018 | Yes | Kennedy SGS, J. J. Morgan, P. J. Peralta, L. R. Hilland, T. A. Eather, N. Lonsdale, C. Okely, A. D. Plotnikoff, R. C. Salmon, J. O. Dewar, D. L. Estabrooks, P. A. Pollock, E. Finn, T. L. Lubans, D. R. Implementing Resistance Training in Secondary Schools: A Cluster Randomized Controlled Trial. Med Sci Sports Exerc. 2018;50(1):62-72. |
| Kipping 2008 | Yes | Kipping RRH, L. D. Jago, R. Campbell, R. Wells, S. Chittleborough, C. R. Mytton, J. Noble, S. M. Peters, T. J. Lawlor, D. A. Effect of intervention aimed at increasing physical activity, reducing sedentary behaviour, and increasing fruit and vegetable consumption in children: active for Life Year 5 (AFLY5) school based cluster randomised controlled trial. BMJ. 2014;348:g3256-g. |
| Kipping 2014 | Yes | Kipping RRP, C. Lawlor, D. A. Randomised controlled trial adapting US school obesity prevention to England. Archives of Disease in Chidhood2008. p. 469-73. |
| Kobel 2017 | Yes | Kobel SL, C. Wartha, O. Kesztyus, D. Wirt, T. Steinacker, J. M. Effects of a Randomised Controlled School-Based Health Promotion Intervention on Obesity Related Behavioural Outcomes of Children with Migration Background. J Immigr Minor Health. 2017;19(2):254-62. |
| Kocken 2016 | Yes | Kocken PLS, A. M. Westhoff, E. De Kok, B. P. Taal, E. M. Goldbohm, R. A. Effects of a Theory-Based Education Program to Prevent Overweightness in Primary School Children. Nutrients. 2016;8(1):04. |
| Kriemler 2010 | Yes | Kriemler SZ, L. Schindler, C. Meyer, U. Hartmann, T. Hebestreit, H. Brunner-La Rocca, H. P. van Mechelen, W. Puder, J. J. Effect of school based physical activity programme (KISS) on fitness and adiposity in primary schoolchildren: cluster randomised controlled trial. BMJ (Clinical research ed). 2010;340:c785-c. |
| Lana 2014 | No | Lana AF-O, G. Lopez, M. L. Impact of a web-based intervention supplemented with text messages to improve cancer prevention behaviors among adolescents: results from a randomized controlled trial. Prev Med. 2014;59:54-9. |
| Lazaar 2007 | Yes | Lazaar NA, J. Ratel, S. Rance, M. Meyer, M. Duché, P. Effect of physical activity intervention on body composition in young children: influence of body mass index status and gender. Acta Paediatrica. 2007;96(9):1315-20. |
| Leme 2016 | Yes | Leme ACL, D. R. Guerra, P. H. Dewar, D. Toassa, E. C. Philippi, S. T. Preventing obesity among Brazilian adolescent girls: Six-month outcomes of the Healthy Habits, Healthy Girls-Brazil school-based randomized controlled trial. Prev Med. 2016;86:77-83. |
| Levy 2012 | Yes | Levy TSMR, C. Amaya Castellanos, C. Salazar Coronel, A. Jiménez Aguilar, A. Méndez Gómez Humarán, I. Effectiveness of a diet and physical activity promotion strategy on the prevention of obesity in Mexican school children. BMC Public Health. 2012;12:152-. |
| Li 2010a | Yes | Li B, Pallan M, Liu WJ, Hemming K, Frew E, Lin R, et al. The CHIRPY DRAGON intervention in preventing obesity in Chinese primary-school--aged children: A cluster-randomised controlled trial. PLoS Med. 2019;16(11):e1002971. |
| Li 2019 | Yes | Li YPH, X. Q. Schouten, E. G. Liu, A. L. Du, S. M. Li, L. Z. Cui, Z. H. Wang, Ly, P. Hu, X. Q. Schouten, E. Report on childhood obesity in China (8): effects and sustainability of physical activity intervention on body composition of Chinese youth. Biomedical & Environmental Sciences. 2010;23(3):180-7. |
| Lichtenstein 2011 | Yes | Lichtenstein STeufel. Prevention of obesity in primary school: A school-based prevention program reduces the risk for obesity in school children. Monatsschrift fur Kinderheilkunde. 2011;159(8):751-7. |
| Liu 2019 | Yes | Liu Z, Li Q, Maddison R, Ni Mhurchu C, Jiang Y, Wei DM, et al. A School-Based Comprehensive Intervention for Childhood Obesity in China: A Cluster Randomized Controlled Trial. Childhood Obesity. 2019;15(2):105-15. |
| Llargues 2012 | Yes | Llargues ER, A. Franco, R. Nadal, A. Vila, M. Perez, M. J. Recasens, I. Salvador, G. Serra, J. Roure, E. Castell, C. Medium-term evaluation of an educational intervention on dietary and physical exercise habits in schoolchildren: the Avall 2 study. Endocrinologia y Nutricion. 2012;59(5):288-95. |
| Lloyd 2018 | Yes | Lloyd JC, S. Logan, S. Green, C. Dean, S. G. Hillsdon, M. Abraham, C. Tomlinson, R. Pearson, V. Taylor, R. S. Ryan, E. Price, L. Streeter, A. Wyatt, K. Effectiveness of the Healthy Lifestyles Programme (HeLP) to prevent obesity in UK primary-school children: a cluster randomised controlled trial. The Lancet Child and Adolescent Health. 2018;2(1):35-45. |
| Lubans 2011 | Yes | Lubans DRM, P. J. Aguiar, E. J. Callister, R. Randomized controlled trial of the Physical Activity Leaders (PALs) program for adolescent boys from disadvantaged secondary schools. Prev Med. 2011;52(3-4):239-46. |
| Luszczynska 2016 | Yes | Luszczynska A, Hagger MS, Banik A, Horodyska K, Knoll N, Scholz U. Self-Efficacy, Planning, or a Combination of Both? A Longitudinal Experimental Study Comparing Effects of Three Interventions on Adolescents' Body Fat. PLoS ONE. 2016;11(7):e0159125. |
| Luszczynska 2016b | No | Luszczynska AH, K. Zarychta, K. Liszewska, N. Knoll, N. Scholz, U. Planning and self-efficacy interventions encouraging replacing energy-dense foods intake with fruit and vegetable: A longitudinal experimental study. Psychol Health. 2016;31(1):40-64. |
| Lynch 2016 | No | Lynch BAG, N. Maxson, J. Quigg, S. Swenson, L. Kaufman, T. Elementary School-Based Obesity Intervention Using an Educational Curriculum. J. 2016;7(4):265-71. |
| Madsen 2015 | Yes | Madsen KL, J. Gerstein, D. Ross, M. Myers, E. Brown, K. Crawford, P. Energy Balance 4 Kids with Play: Results from a Two-Year Cluster-Randomized T{Morgan, 2014 #45}rial. Childhood Obesity. 2015;11(4):375-83. |
| Magnusson 2012 | Yes | Magnusson KTMK, Thor Hrafnkelsson, Hannes Sigurgeirsson, Ingvar Johannsson, Erlingur Sveinsson, T. h o r a r i n n. Limited effects of a 2-year school-based physical activity intervention on body composition and cardiorespiratory fitness in 7-year-old children. Health Educ Res. 2012;27(3):484-94. |
| Marcus 2009 | No | Marcus CN, G. Nordenfelt, A. Karpmyr, M. Kowalski, J. Ekelund, U. A 4-year, cluster-randomized, controlled childhood obesity prevention study: STOPP. International Journal of Obesity2009. p. 408-17. |
| Mauriello 2010 | No | Mauriello LMC, M. M. Paiva, A. L. Sherman, K. J. Castle, P. H. Johnson, J. L. Prochaska, J. M. Results of a multi-media multiple behavior obesity prevention program for adolescents. Prev Med. 2010;51(6):451-6. |
| Melnyk 2013 | Yes | Melnyk BMJ, D. Kelly, S. Belyea, M. Shaibi, G. Small, L. O'Haver, J. Marsiglia, F. F. Promoting healthy lifestyles in high school adolescents: a randomized controlled trial. Am J Prev Med. 2013;45(4):407-15. |
| Meng 2020 | Yes | Meng Y, Lohse B, Cunningham-Sabo L. Sex modifies the association between the CLOCK variant rs1801260 and BMI in school-age children. PLoS ONE. 2020;15(8):e0236991. |
| Mihas 2010 | Yes | Mihas CM, A. Manios, Y. Naska, A. Arapaki, A. Mariolis-Sapsakos, T. Tountas, Y. Evaluation of a nutrition intervention in adolescents of an urban area in Greece: short- and long-term effects of the VYRONAS study. Public Health Nutr. 2010;13(5):712-9. |
| Muckelbauer 2010 | No | Muckelbauer RL, L. Clausen, K. Toschke, A. Immigrational background affects the effectiveness of a school-based overweight prevention program promoting water consumption. Obesity. 2010;18(3):528-34. |
| Muller 2016 | No | Muller I, Schindler C, Adams L, Endes K, Gall S, Gerber M, et al. Effect of a Multidimensional Physical Activity Intervention on Body Mass Index, Skinfolds and Fitness in South African Children: Results from a Cluster-Randomised Controlled Trial. Int J Environ Res Public Health. 2019;16(2):15. |
| Muller 2019 | Yes | Muller UMW, C. Adams, V. Mende, M. Adam, J. Fikenzer, K. Machalica, K. C. Erbs, S. Linke, A. Schuler, G. Long term impact of one daily unit of physical exercise at school on cardiovascular risk factors in school children. European Journal of Preventive Cardiology. 2016;23(13):1444-52. |
| Neumark-Sztainer 2003 | No | Neumark-Sztainer DRF, S. E. Flattum, C. F. Hannan, P. J. Story, M. T. Bauer, K. W. Feldman, S. B. Petrich, C. A. New moves-preventing weight-related problems in adolescent girls a group-randomized study. Am J Prev Med. 2010;39(5):421-32. |
| Neumark-Sztainer 2010 | No | Neumark-Sztainer DS, M. Hannan, P. J. Rex, J. New Moves: a school-based obesity prevention program for adolescent girls. Prev Med. 2003;37(1):41-51. |
| Nyberg 2015 | No | Nyberg GS, E. Norman, A. Bohman, B. Hagberg, J. Elinder, L. S. Effectiveness of a universal parental support programme to promote healthy dietary habits and physical activity and to prevent overweight and obesity in 6-year-old children: the Healthy School Start Study, a cluster-randomised controlled trial. PLoS ONE. 2015;10(2):e0116876. |
| Pate 2005 | No | Pate RRW, D. S. Saunders, R. P. Felton, G. Dishman, R. K. Dowda, M. Promotion of physical activity among high-school girls: a randomized controlled trial. Am J Public Health. 2005;95(9):1582-7. |
| Peralta 2009 | Yes | Peralta LRJ, R. A. Okely, A. D. Promoting healthy lifestyles among adolescent boys: the Fitness Improvement and Lifestyle Awareness Program RCT. Prev Med2009. p. 537-42. |
| Polonsky 2019 | Yes | Polonsky HM, Bauer KW, Fisher JO, Davey A, Sherman S, Abel ML, et al. Effect of a Breakfast in the Classroom Initiative on Obesity in Urban School-aged Children: A Cluster Randomized Clinical Trial. Jama, Pediatr. 2019;173(4):326-33. |
| Ramirez-Rivera 2021 | Yes | Ramirez-Rivera DL, Martinez-Contreras T, Villegas-Valle RC, Henry-Mejia G, Quizan-Plata T, Haby MM, et al. Preliminary Results of the Planet Nutrition Program on Obesity Parameters in Mexican Schoolchildren: Pilot Single-School Randomized Controlled Trial. Int J Environ Res Public Health. 2021;18(2):18. |
| Reed 2008 | Yes | Reed KEW, D. E. Macdonald, H. M. Naylor, P. J. McKay, H. A. Action Schools! BC: a school-based physical activity intervention designed to decrease cardiovascular disease risk factors in children. Prev Med. 2008;46(6):525-31. |
| Rerksuppaphol 2017 | Yes | Rerksuppaphol LR, S. Internet Based Obesity Prevention Program for Thai School Children- A Randomized Control Trial. J Clin Diagn Res. 2017;11(3):SC07-SC11. |
| Robbins 2006 | Yes | Robbins LBG, K. A. Kazanis, A. S. Pender, N. J. Girls on the move program to increase physical activity participation. Nursing Research. 2006;55(3):206-16. |
| Rosario 2012 | Yes | Rosario RO, B. Araujo, A. Lopes, O. Padrao, P. Moreira, A. Teixeira, V. Barros, R. Pereira, B. Moreira, P. The impact of an intervention taught by trained teachers on childhood overweight. International Journal of Environmental Research & Public Health. 2012;9(4):1355-67. |
| Rush 2012 | No | Rush ER, P. McLennan, S. Coppinger, T. Simmons, D. Graham, D. A school-based obesity control programme: Project Energize. Two-year outcomes. Br J Nutr. 2012;107(4):581-7. |
| Safdie 2013 | Yes | Safdie MJ-A, N. Levesque, L. Janssen, I. Campirano-Nunez, F. Lopez-Olmedo, N. Aburto, T. Rivera, J. A. Impact of a school-based intervention program on obesity risk factors in Mexican children. Salud Publica Mex. 2013;55(Suppl 3):374-87. |
| Sahota 2001 | Yes | Sahota P, Christian M, Day R, Cocks K. The feasibility and acceptability of a primary school-based programme targeting diet and physical activity: the PhunkyFoods Programme. Pilot and feasibility studies. 2019;5(1). |
| Sahota 2019 | Yes | Sahota PR, M. C. J. Dixey, R. Hill, A. J. Barth, J. H. Cade, J. Evaluation of implementation and effect of primary school based intervention to reduce risk factors for obesity. BMJ. 2001;323:1027-9. |
| Sallis 1993 | No | Sallis JFM, T. L. Alcaraz, J. E. Kolody, B. Hovell, M. F. Nader, P. R. Project SPARK. Effects of physical education on adiposity in children. Annals of the New York Academy of Sciences. 1993;699:127-36. |
| Salmon 2008 | No | Salmon JB, K. Hume, C. Booth, M. Crawford, D. Outcomes of a group-randomized trial to prevent excess weight gain, reduce screen behaviours and promote physical activity in 10-year-old children: switch-play. International Journal of Obesity (Lond)2008. p. 601-12. |
| Santos 2014 | Yes | Santos RGD, A. Rabbanni, R. Chanoine, J. P. Lamboo Miln, A. Mayer, T. McGavock, J. M. Effectiveness of peer-based healthy living lesson plans on anthropometric measures and physical activity in elementary school students: a cluster randomized trial.[Erratum appears in JAMA Pediatr. 2015 Jan;169(1):96]. Jama, Pediatr. 2014;168(4):330-7. |
| Sevinc 2011 | Yes | Sevinc OB, A. I. Gundogdu, M. Bas Aslan, U. Agbuga, B. Aslan, S. Dikbas, E. Gokce, Z. Evaluation of the effectiveness of an intervention program on preventing childhood obesity in Denizli, Turkey. Turkish Journal of Medical Sciences. 2011;41(6):1097-105. |
| Sgambato 2019 | No | Sgambato MR, Cunha DB, da Silva Nalin Souza B, Henriques VT, da Rocha Muniz Rodrigues R, Viegas Rego AL, et al. Effectiveness of school-home intervention for adolescent obesity prevention: parallel school-randomized study. Br J Nutr. 2019:1-20. |
| Sichieri 2008 | Yes | Sichieri RPT, A. de Souza, R. A. Veiga, G. V. School randomised trial on prevention of excessive weight gain by discouraging students from drinking sodas. Public Health Nutr2008. p. 197-202. |
| Siegrist 2013 | Yes | Siegrist M, Hanssen H, Lammel C, Haller B, Koch AM, Stemp P, et al. Effects of a cluster-randomized school-based prevention program on physical activity and microvascular function (JuvenTUM 3). Atherosclerosis. 2018;278:73-81. |
| Siegrist 2018 | Yes | Siegrist ML, C. Haller, B. Christle, J. Halle, M. Effects of a physical education program on physical activity, fitness, and health in children: the JuvenTUM project. Scandinavian Journal of Medicine & Science in Sports. 2013;23(3):323-30. |
| Simon 2008 | Yes | Simon CS, B. Oujaa, M. Wagner, A. Arveiler, D. Triby, E. et, a l. Successful overweight prevention in adolescents by increasing physical activity: a 4-year randomized controlled intervention. International Journal of Obesity (Lond)2008. p. 1489-98. |
| Singh 2009 | Yes | Singh ASCAP, M. J. Brug, J. Van Mechelen, W. Dutch obesity intervention in teenagers: effectiveness of a school-based program on body composition and behavior. Archives of Pediatrics & Adolescent Medicine2009. p. 309-17. |
| Smith 2014 | Yes | Smith JJM, P. J. Plotnikoff, R. C. Dally, K. A. Salmon, J. Okely, A. D. Finn, T. L. Lubans, D. R. Smart-phone obesity prevention trial for adolescent boys in low-income communities: the ATLAS RCT. Pediatrics. 2014;134(3):e723-e31. |
| Spiegel 2006 | No | Spiegel SAF, D. Reducing overweight through a multidisciplinary school-based intervention. Obesity. 2006;14(1):88-96. |
| Telford 2012 | No | Telford RDC, R. B. Fitzgerald, R. Olive, L. S. Prosser, L. Jiang, X. Telford, R. M. Physical education, obesity, and academic achievement: a 2-year longitudinal investigation of Australian elementary school children. Am J Public Health. 2012;102(2):368-74. |
| TenHoor 2018 | No | Ten Hoor GA, Rutten GM, Van Breukelen GJP, Kok G, Ruiter RAC, Meijer K, et al. Strength exercises during physical education classes in secondary schools improve body composition: a cluster randomized controlled trial. Int. 2018;15(1):92. |
| Thivel 2011 | Yes | Thivel DI, L. Lazaar, N. Aucouturier, J. Ratel, S. Dore, E. Meyer, M. Duche, P. Effect of a 6-month school-based physical activity program on body composition and physical fitness in lean and obese schoolchildren. European Journal of Pediatrics. 2011;170(11):1435-43. |
| Treviño 2004 | No | Treviño RP, Yin Z, Hernandez A, Hale DE, Garcia OA, Mobley C. Impact of the Bienestar school-based diabetes mellitus prevention program on fasting capillary glucose levels: a randomized controlled trial. Arch Pediatr Adolesc Med. 2004;158(9):911-7. |
| Velez 2010 | Yes | Velez AG, D. L. Arent, S. M. The impact of a 12-week resistance training program on strength, body composition, and self-concept of Hispanic adolescents. Journal of Strength & Conditioning Research. 2010;24(4):1065-73. |
| Viggiano 2015 | Yes | Viggiano AV, E. Di Costanzo, A. Viggiano, A. Andreozzi, E. Romano, V. Rianna, I. Vicidomini, C. Gargano, G. Incarnato, L. Fevola, C. Volta, P. Tolomeo, C. Scianni, G. Santangelo, C. Battista, R. Monda, M. Viggiano, A. De Luca, B. Amaro, S. Kaledo, a board game for nutrition education of children and adolescents at school: cluster randomized controlled trial of healthy lifestyle promotion. European Journal of Pediatrics. 2015;174(2):217-28. |
| Viggiano 2018 | Yes | Viggiano E, Viggiano A, Di Costanzo A, Viggiano A, Viggiano A, Andreozzi E, et al. Healthy lifestyle promotion in primary schools through the board game Kaledo: a pilot cluster randomized trial. European Journal of Pediatrics. 2018;177(9):1371-5. |
| Wang 2012 | No | Wang YY, Y. Xu, G. F. Evaluating the effect of school-based children obesity prevention and control. Chinese Health Service Management. 2012;4:317-9. |
| Wang 2018 | Yes | Wang Z, Xu F, Ye Q, Tse LA, Xue H, Tan Z, et al. Childhood obesity prevention through a community-based cluster randomized controlled physical activity intervention among schools in china: the health legacy project of the 2nd world summer youth olympic Games (YOG-Obesity study). International Journal of Obesity. 2018;42(4):625-33. |
| Warren 2003 | No | Warren JMH, C. J. K. Lightowler, H. J. Bradshaw, S. M. Perwaiz, S. Evaluation of a pilot school programme aimed at the prevention of obesity in children. Health Promotion International. 2003;18(4):287-96. |
| Waters 2017 | No | Waters E, Gibbs L, Tadic M, Ukoumunne OC, Magarey A, Okely AD, et al. Cluster randomised trial of a school-community child health promotion and obesity prevention intervention: findings from the evaluation of fun 'n healthy in Moreland! BMC Public Health. 2017;18(1):92. |
| Weeks 2012 | Yes | Weeks BKB, B. R. Twice-weekly, in-school jumping improves lean mass, particularly in adolescent boys. Pediatr Obes. 2012;7(3):196-204. |
| Wendel 2016 | Yes | Wendel MLB, M. E. Zhao, H. Jeffrey, C. Stand-Biased Versus Seated Classrooms and Childhood Obesity: A Randomized Experiment in Texas. Am J Public Health. 2016;106(10):1849-54. |
| Whittemore 2013 | Yes | Whittemore RJ, S. Grey, M. An internet obesity prevention program for adolescents. Journal of adolescent health. 2013;52(4):439-47. |
| Wilksch 2015 | Yes | Wilksch SMP, S. J. Byrne, S. M. Austin, S. B. McLean, S. A. Thompson, K. M. Dorairaj, K. Wade, T. D. Prevention Across the Spectrum: a randomized controlled trial of three programs to reduce risk factors for both eating disorders and obesity. Psychol Med. 2015;45(9):1811-23. |
| Williamson 2012 | Yes | Williamson DAC, C. M. Harsha, D. W. Han, H. Martin, C. K. Newton Rl, J. r Sothern, M. S. Stewart, T. M. Webber, L. S. Ryan, D. H. Effect of an environmental school-based obesity prevention program on changes in body fat and body weight: a randomized trial. Obesity. 2012;20(8):1653-61. |
| Xu 2015 | Yes | Xu FW, R. S. Leslie, E. Tse, L. A. Wang, Z. Li, J. Wang, Y. Effectiveness of a Randomized Controlled Lifestyle Intervention to Prevent Obesity among Chinese Primary School Students: CLICK-Obesity Study. PLoS ONE. 2015;10(10):e0141421. |
| Xu 2017 | Yes | Xu HL, Y. Zhang, Q. Hu, X. L. Liu, A. Du, S. Li, T. Guo, H. Li, Y. Xu, G. Liu, W. Ma, J. Ma, G. Comprehensive school-based intervention to control overweight and obesity in China: a cluster randomized controlled trial. Asia Pac J Clin Nutr. 2017;26(6):1139-51. |
| Zhou 2019 | No | Zhou Z, Li S, Yin J, Fu Q, Ren H, Jin T, et al. Impact on Physical Fitness of the Chinese CHAMPS: A Clustered Randomized Controlled Trial. Int J Environ Res Public Health. 2019;16(22):11. |
| Zota 2016 | No | Zota DD, A. Petralias, A. Lykou, A. Kastorini, C. M. Yannakoulia, M. Karnaki, P. Belogianni, K. Veloudaki, A. Riza, E. Malik, R. Linos, A. Promotion of healthy nutrition among students participating in a school food aid program: a randomized trial. Int J Public Health. 2016;61(5):583-92. |
