# Supplementary material for: Identifying behaviour change techniques in school-based childhood obesity prevention interventions: a secondary analysis of a systematic review
Source: BMC Public Health. 2025 Jul 2;25:2250. doi: 10.1186/s12889-025-23421-9 (PMC12219750; doi:10.1186/s12889-025-23421-9)
Supplement: Supplementary file 5 [file 12889_2025_23421_MOESM5_ESM.docx]

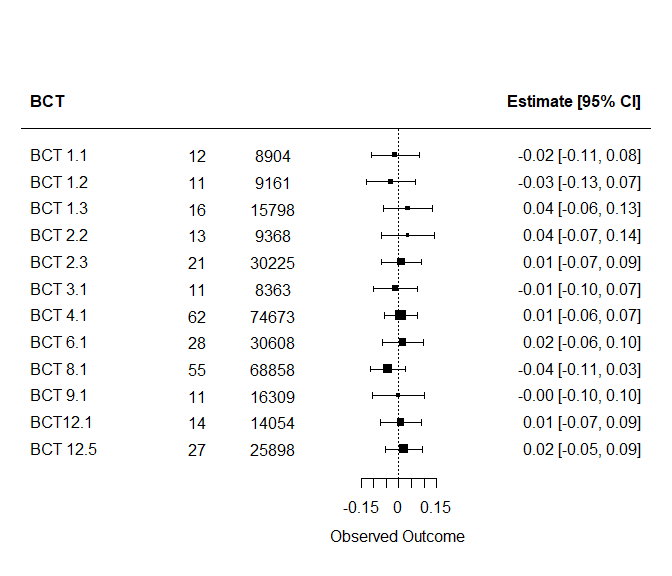


**Additional file 5:** Meta-regression showing effects of individual BCTs in physical activity interventions on BMI/BMI z-scores. Legend: *BCT 1.1 Goal setting (behaviour); 1.2 Problem solving; 1.3 Goal setting (outcome); 2.2 Feedback on behaviour; 2.3 Self-monitoring of behaviour; 3.1 Social support (unspecified); 4.1 Instructions on how to perform the behaviour; 6.1 Demonstration of the behaviour; 8.1 Behavioural practice/rehearsal; 9.1 Credible source; 12.1 Restructuring the physical environment; 12.5 Adding objects to the environment*
