# Supplementary material for: Identifying behaviour change techniques in school-based childhood obesity prevention interventions: a secondary analysis of a systematic review
Source: BMC Public Health. 2025 Jul 2;25:2250. doi: 10.1186/s12889-025-23421-9 (PMC12219750; doi:10.1186/s12889-025-23421-9)
Supplement: Supplementary file 7 [file 12889_2025_23421_MOESM7_ESM.docx]

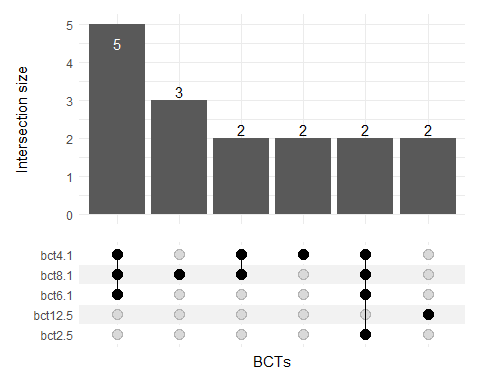


*

**Additional file 7:** Upset plot showing the discrete individual BCTs or combinations of BCTs used in PA interventions. Legend: *Intersection size = the number of studies that used the specific combination of BCTs listed below the column.
